# Accurate determination of causalities in gene regulatory networks by dissecting downstream target genes

**DOI:** 10.3389/fgene.2022.923339

**Published:** 2022-12-07

**Authors:** Zhigang Jia, Xiujun Zhang

**Affiliations:** ^1^ School of Mathematics and Statistics, Xinyang Normal University, Xinyang, China; ^2^ Key Laboratory of Plant Germplasm Enhancement and Specialty Agriculture, Wuhan Botanical Garden, Chinese Academy of Sciences, Wuhan, China; ^3^ Center of Economic Botany, Core Botanical Gardens, Chinese Academy of Sciences, Wuhan, China

**Keywords:** gene regulatory networks, network inference, downstream targets, causality, machine learning

## Abstract

Accurate determination of causalities between genes is a challenge in the inference of gene regulatory networks (GRNs) from the gene expression profile. Although many methods have been developed for the reconstruction of GRNs, most of them are insufficient in determining causalities or regulatory directions. In this work, we present a novel method, namely, DDTG, to improve the accuracy of causality determination in GRN inference by dissecting downstream target genes. In the proposed method, the topology and hierarchy of GRNs are determined by mutual information and conditional mutual information, and the regulatory directions of GRNs are determined by Taylor formula-based regression. In addition, indirect interactions are removed with the sparseness of the network topology to improve the accuracy of network inference. The method is validated on the benchmark GRNs from DREAM3 and DREAM4 challenges. The results demonstrate the superior performance of the DDTG method on causality determination of GRNs compared to some popular GRN inference methods. This work provides a useful tool to infer the causal gene regulatory network.

## Introduction

Elucidating gene regulatory networks (GRNs) is a fundamental challenge in molecular biology (Hughes et al., 2000). High-throughput technologies provided a wealth of gene expression data which are helpful to interrogate the complex regulatory dynamics inherent to organisms (Algabri et al., 2022; Wang and Liu, 2022). The network structure with genes (genes) and regulatory interactions (edges) can be inferred from the observed data through minimizing the effects of noise and hidden genes (Baruch and Albert-László, 2013; Yang et al., 2022). To improve the accuracy of network reconstruction, various methods have been developed for the reconstruction of GRNs from gene expression profiles (Riet and Kathleen, 2010; Zhang et al., 2022). However, each method has its own strengths and weaknesses (Daniel et al., 2010). Among the methods for GRN inference, most of them are insufficient in determining the causalities or regulatory directions (Krouk et al., 2013; Ahmed et al., 2018). Understanding the causality in the gene expression data is critical to elucidating the underlying regulatory mechanism of cellular machines (Jiang et al., 2000; Nagoshi et al., 2004; Rubin et al., 2019).

Existing methods to infer the GRNs from gene expression data with the motivation of improving the accuracy and scalability of network inference include model-based approaches and machine learning-based approaches (Madar et al., 2009; Zhang et al., 2013). For the model-based approaches, chemical reaction of transcription and translation, as well as other cellular processes, are described as linear or nonlinear differential equations, in which the parameters represent the regulation strengths of the regulators (Gardner et al., 2003; Honkela et al., 2010). Dynamical system models of differential equations can forecast future system behaviors and characterize formal properties such as stability (Zak et al., 2003; Wang et al., 2022). Furthermore, prior information, such as experimentally verified regulations, can be easily included in these models to improve the accuracy of network inference (Studham et al., 2014; Zhang et al., 2017). Moreover, the model-based methods are found useful to remove possible redundant indirect regulations by forcing sparseness on the model (Hurley et al., 2011; Jiang and Zhang, 2022). However, these models are computationally intractable for large GRNs owing to extensive and explicit parameterization requirements (Karlebach and Shamir, 2008; Tibshirani, 2011). For the machine learning-based approaches, the network is inferred through measuring the dependences or causalities between transcriptional factors and target genes (Khatamian et al., 2018; Deng et al., 2021). Popular methods in this category include mutual information (MI) (Modi et al., 2011), conditional mutual information (CMI) (Zhang et al., 2011), part mutual information (Zhao et al., 2016), Granger causality (Finkle et al., 2018), and maximal information coefficient (Reshef et al., 2011; Kinney and Atwal, 2014). With no explicit mechanistic assumptions, the machine learning-based methods are usually more efficient than the model-based methods in the computational complex (Zhang et al., 2015).

As the most popular methods, MI and CMI evaluate the association between the genes by measuring the entropy of their mutual activities, where a lower entropy for a gene indicates that its activities are less randomly distributed; that is, it is statistically dependent on the activities of other genes (Butte and Kohane, 2000). Specially, MI can characterize nonlinear dependency and deal with thousands of variables (genes) in the presence of a limited number of samples. However, the MI between two variables is a symmetric quantity, and so the MI-based methods generally infer undirected interactions (Aghdam et al., 2015). The ordinary differential equation (ODE)-based methods can be used for inference of causal GRNs, but these methods require rigorous datasets like time-series data (Lu et al., 2021; Yang et al., 2021; Chen and Liu, 2022).

In this study, we develop a method for inferring causal GRNs from genetic knockout data. The activities of the downstream target genes respond to the knockout changes of regulatory genes and are identified accurately by comparing the relative change values of the downstream targets and assigning a weight to the relative change values of each gene. It is helpful to remove the spurious edges in the causal inference of GRNs. We dissect the downstream target genes using CMI, MI, and Taylor formula-based regression to determine the causalities among the downstream targets. We model the hierarchy structure of the downstream targets and determine the causalities in different layers with CMI which is efficient to remove these redundant indirect regulations. Consequently, we determine the correlation in the same layer. At last, we use Taylor formula-based regression to determine the causalities in the same layer. With the process repeating for each regulator, the reconstruction of GRNs is achieved. Our method not only has the advantages of the machine learning-based methods but also can determine the regulatory directions. The results on the DREAM3 and DREAM4 datasets show that our method significantly outperforms the existing method in terms of false-positive rates and accuracy.

## Methods

### Downstream target identification

As the activity of the regulator can be approximated by the expression level of the gene encoding the regulator, we suppose the gene expression level as regulator activity. Let $g_{i}$ represent the *i*th gene. Considering a network consisted of *n* genes, gene expression matrix *A* denotes the gene expression level under different conditions (samples) which can be measured directly from gene knockout experiments. The knockout experiment is implemented for every gene and the downstream gene response to the knockout of gene through the fluctuations of expression levels. The steady-state levels of genes in the wild-type provide a standard of the gene expression changes. Thus, the gene expression levels of wild-type and knockout experiments of each gene provide the information to identify downstream targets. Matrix *A* consists of *n* rows with *n* steady-state values of knockout experiments, and each row is obtained after deleting one of *n* genes. The vector $A_{j}={\left(a_{1j},a_{2j,}\cdots a_{nj}\right)}^{T}$ stands for the *j*th column of matrix *A*, in which $a_{ij}$ represents the steady-state level of gene $g_{j}$ after knockout of gene $g_{i}$ . The wild-type file contained *n* steady-state levels of the unperturbed network. The vector $A_{0}=\left(a_{01},a_{02},\cdots ,a_{0n}\right)$ stands for the wild-type data of each gene, in which $a_{0j}$ denotes the steady-state level of the wild-type of gene $g_{j}$ .

**FIGURE 1:**
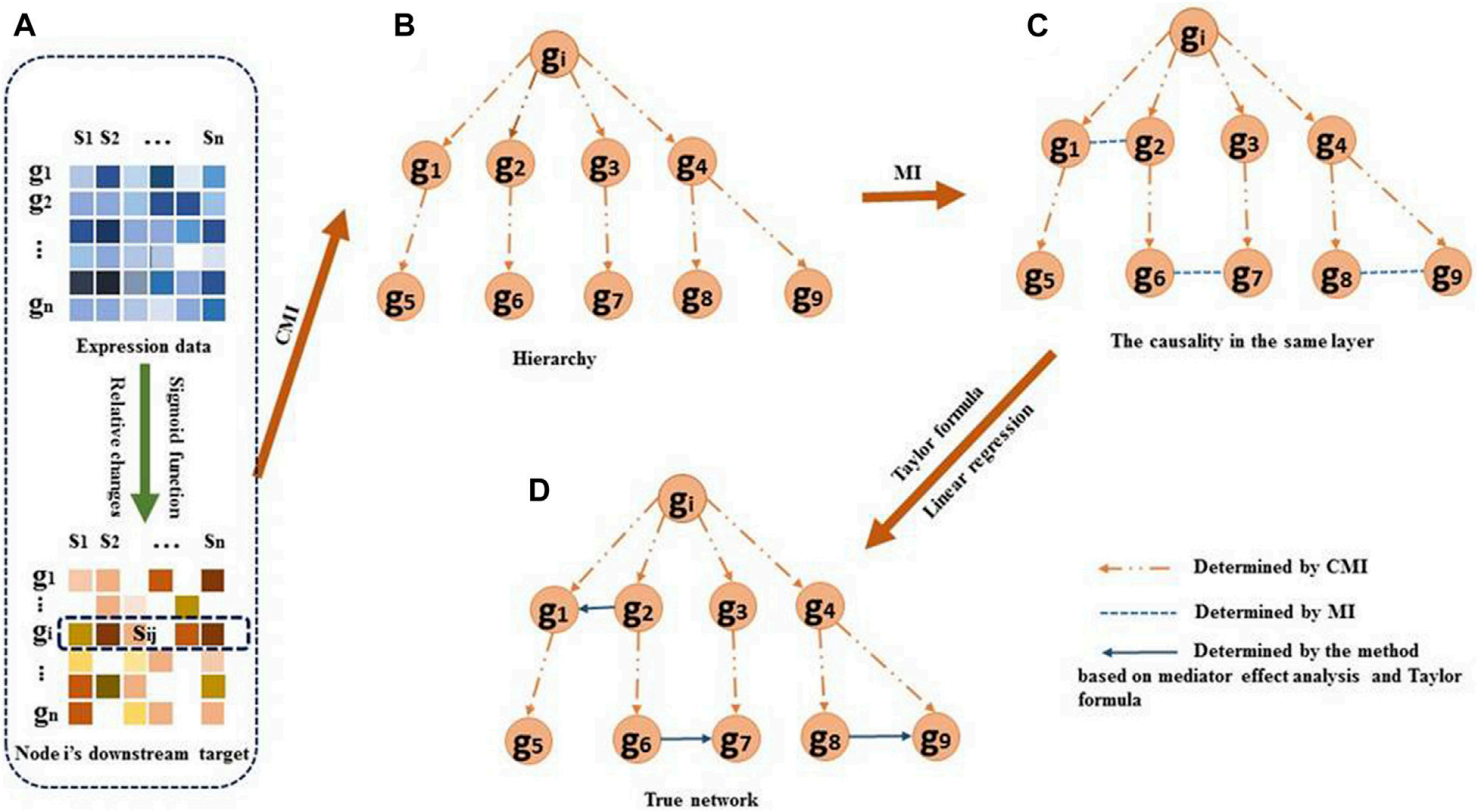
Overview of the DDTG method. **(A)** For each regulator $g_{i}$ , the downstream target genes $s_{ij}$ of the regulator are identified by the Sigmoid function and by comparing the relative change values. **(B)** Hierarchy structure of downstream target genes will be decided by using CMI. Assuming genes $g_{1}–g_{9}$ are the downstream target genes of regulator $g_{i}$ . As an example, genes $g_{1}$ and $g_{5}$ belong to the 2 combination of the downstream target genes. If $I\left(g_{i},g_{5}|g_{1}\right)$ is small, it indicates that $g_{i}$ regulates $g_{1}$ directly and $g_{1}$ regulates $g_{5}$ directly. The dashed arrows denote the regulatory direction. **(C)** The correlations between two genes in the same layer. The dashed lines without arrows denote the genes being strongly dependent on each other. Edges $g_{1}$ – $g_{2}$ , $g_{6}$ – $g_{7}$ , and $g_{8}$ – $g_{9}$ are directly correlated with each other in the same layer. **(D)** The regulatory direction between two genes in the same layer is determined using the Taylor formula and linear regression. The solid arrows denote the causality in the same layer. The reconstruction of GRNs is achieved by aggregating the edges from each step.

The genes whose steady-state values change as a result of a single-gene knockout are likely to be downstream of the perturbed gene. Most causal relationships owing to the gene knockout could be immediately recognized by 0comparing the steady-state data after gene knockout with wild-type data. We calculate the changes of gene $g_{j}$ response to the knockout of every gene by using the following expression:

$$\Delta A_{j}={\left(\Delta a_{ij}\right)}^{T}={\left(a_{1j}-a_{0j},a_{2j}-a_{0j},\cdots ,a_{nj}-a_{0j}\right)}^{T},$$ (1)

where $\Delta a_{ij}=a_{ij}-a_{0j}$ denotes the changes of gene $g_{j}$ by comparing the response to the knockout of gene $g_{i}$ with the wild type. The changes describe the response of all genes as a consequence of the perturbation of the source gene. We use the mean change value to quantify the mean response strengths of the same target to different regulators. The mean change value in gene $g_{j}$ can be expressed as $\bar{\Delta A_{j}}=\frac{1}{n}\overset{n}{\underset{i=1}{\sum }}\left|\Delta a_{ij}\right|$ . $\Delta a_{ij}$ for different genes varies widely because the wild-type data of different genes vary widely. So we use the relative change value to quantify the response strengths of the same target to different regulators. We obtain the relative change value vector which is $∆S_{\cdot j}={\left(∆s_{1j},∆s_{2j}\cdots ∆s_{nj}\right)}^{T}$ , where $∆s_{ij}=∆a_{ij}/\bar{∆A_{j}}$ denotes a relative change value of gene $g_{j}$ . Gene $g_{i}$ is called as the regulator, and the genes that respond to the change of $g_{i}$ are called downstream target genes or targets. $a_{ij}-a_{0j}$ of each gene varies widely because the wild-type data of each gene vary widely and because the activities of the downstream target genes responding to the same knock-out change of regulatory gene vary widely. To calculate the activities of the downstream target genes and compare the relative changes of $g_{j}$ with other genes, we assign a weight to $∆S_{\cdot \;j}$ by sigmoid function $w_{j}=1/\left(1+e^{r\left(b_{j}-u\right)}\right)$ , where parameters *r* and *u* describe the coefficients of sigmoid function, and $b_{j}=\max_{i}\left|∆a_{ij}\right|/a_{0j}$ describes the maximum response strength of $g_{j}$ to the changes of other genes. Parameters *r* and *u* are given but not estimated to balance the computation of *w*. Parameter *r* is set as a negative integer number and parameter *u* is set as a positive number and is smaller than 1. In general, the values chosen will not affect the final results. By calculating $S_{\cdot \;j}=w_{j}∆S_{\cdot \;j}$ , we obtain a matrix $S={\left(S_{i,j}\right)}_{n\times n}$ , where $S_{\cdot j}$ denotes the j th column of matrix *S*, and the row vector $S_{i\;\cdot }$ denotes the *i*th row of matrix *S*. Given a threshold parameter $\theta_{0}$ for deciding the downstream target genes of regulator $g_{i}$ , the elements in $S_{i\;\cdot }$ above $\theta_{0}$ are regarded as downstream target genes $g_{i}$ (Figure 1A). Most casual relationships could be accurately recognized from $S_{i\;\cdot }$ . Due to the sparseness of GRNs, the downstream targets consist of a small number of genes, which is helpful to remove the indirect dependencies and reduce the computational complexity.

### Causality among hierarchy genes

Some of the downstream targets may be indirectly regulated by gene $g_{i}$ . The remaining task is thus to distinguish direct dependence from indirect dependence. To accomplish this, we use conditional mutual information (CMI) to discriminate the genes directly regulated by $g_{i}$ from the genes indirectly regulated by $g_{i}$ . Accordingly, we obtain a hierarchy structure of the downstream targets of $g_{i}$ . So the topological structure of the downstream target genes of $g_{i}$ is a two-layer network. The genes in the first layer are directly regulated by regulator $g_{i}$ , and the genes in the second layer are indirectly regulated by $g_{i}$ .

The CMI allows measuring the dependency of two genes in the context of a third gene. We assume that $g_{j}$ and $g_{k}$ are $g_{i}$ ’s downstream target genes. The interaction between gene $g_{i}$ and $g_{j}$ can be measured in the context of gene $g_{k}$ by the CMI, which is defined as follows:

$$I\left(g_{i},g_{j}|g_{k}\right)=\underset{x\in g_{i},\;y\in g_{j},\;z\in g_{k}}{\sum }p\left(x,y,z\right)\log\frac{p\left(x,y|z\right)}{p\left(x|z\right)p\left(y|z\right)}$$

The CMI can be easily calculated using the following equation:

$$I\left(g_{i},g_{j}|g_{k}\right)=\frac{1}{2}\log\frac{\left|C\left(g_{i},g_{k}\right)\right|\cdot \left|C\left(g_{j},g_{k}\right)\right|}{\left|C\left(g_{k}\right)\right|\cdot \left|C\left(g_{i},g_{j},g_{k}\right)\right|},$$ (2)

where C is the covariance matrix of variables, and $\left|C\right|$ is the determinant of matrix C . If $g_{j}$ and $g_{k}$ carry the same information about $g_{i}$ , $I\left(g_{i},g_{j}|g_{k}\right)=0$ . It indicates that $g_{i}$ directly regulates $g_{k}$ and $g_{i}$ indirectly regulates $g_{j}$ mediated by $g_{k}$ ; that is, gene $g_{k}$ directly regulates gene $g_{j}$ . The genes regulated directly by $g_{i}$ form a layer, namely, the first layer, and the genes regulated indirectly by $g_{i}$ form a layer, namely, the second layer (Figure 1B).

### Correlations among the genes in the same layer

For the genes in the same layer, the correlations among them are measured by mutual information (MI). MI between two genes $g_{h}$ and $g_{l}$ can be defined as follows (Altay and Emmert-Streib, 2010):

$$I\left(g_{h},g_{l}\right)=-\underset{x\in g_{k},y\in g_{l}}{\sum }p\left(x,y\right)\log\frac{p\left(x,y\right)}{p\left(x\right)p\left(y\right)}$$

The MI can be easily calculated using the following equivalent formula:

$$I\left(g_{h},g_{l}\right)=\frac{1}{2}\log\frac{\left|C\left(g_{h}\right)\right|\cdot \left|C\left(g_{l}\right)\right|}{\left|C\left(g_{h},g_{l}\right)\right|},$$ (3)

where C is the covariance matrix of variables, and $\left|C\right|$ is the determinant of matrix C . If $I\left(g_{h},g_{l}\right)$ is large, it indicates that a strong dependency exists between genes $g_{h}$ and $g_{l}$ (Figure 1C).

### Regulatory directions between genes in the same layer

To determine if the regulatory direction in the scenario of $g_{h}$ and $g_{l}$ is in the same layer and a strong dependency exists between them, we here propose an innovative and effective approach based on linear regression.

**FIGURE 2:**
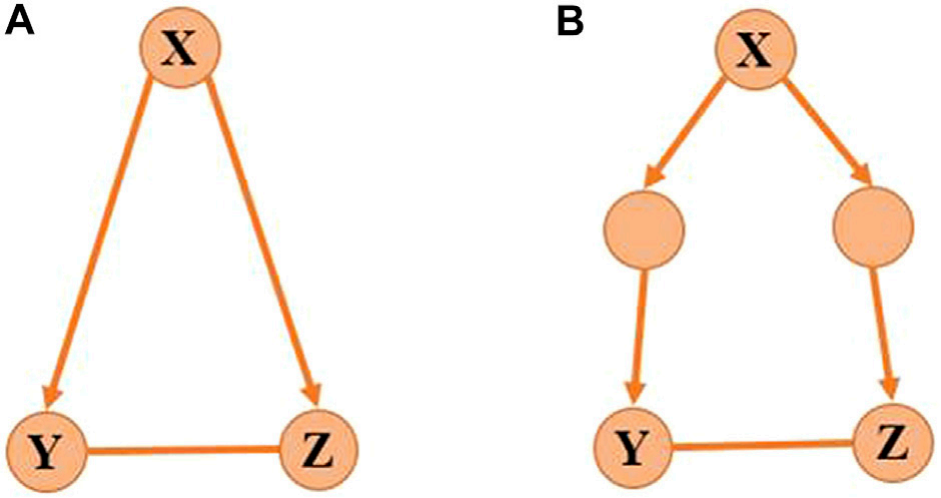
Target genes with co-regulator. **(A)** Genes *Y* and *Z* are direct targets of gene *X*, and **(B)** genes *Y* and *Z* are indirect targets of gene *X.*

We assume that gene $g_{m}$ is the common regulator of genes $g_{h}$ and $g_{l}$ , and that a strong dependency exists between gene $g_{h}$ and gene $g_{l}$ by measuring the MI. We denote $g_{m}$ , $g_{h}$ , and $g_{l}$ by *X*, *Y*, and *Z*, respectively, for simplifying notations. We aim to determine the regulatory direction between *Y* and *Z* in the direct gene set (Figure 2A) or the indirect gene set (Figure 2B). If we assume gene *Y* regulates gene *Z*, the gene–gene regulation can be expressed as a nonlinear equation set:

$$\left\{\begin{array}{l} Y=f\left(X\right),\\ Z=g\left(X,Y\right)=g\left(X,f\left(X\right)\right)=h\left(X\right). \end{array}\right.$$ (4)

The activity of *Y* is determined by *X* and the activity of *Z* is determined by *X* and *Y.* So *Y* will be the function with respect to *X*, and *Z* will be the function with respect to *X* and *Y* satisfying Eq. 4 which indicates the causality among *X*, *Y*, and *Z*. The nonlinear regulatory function 4) makes it difficult to computationally identify the model. To address this issue, we apply Taylor expansion which is an accurate substitution of the polynomial function for the nonlinear equation Eq. 4.If $X_{0}$ , $Y_{0}$ , and $Z_{0}$ denote the wild-type data of *X*, *Y*, and *Z*, then $Y_{0}=f\left(X_{0}\right)$ and $Z_{0}=g\left(X_{0}\right)$ . The Taylor expansion corresponding to $Y=f\left(X\right)$ and $Z=h\left(X\right)$ at point $X_{0}$ is the infinite series whose *n*th term is $h^{\prime }\left(x_{0}\right){\left(x-x_{0}\right)}^{n}/n!$ , that is,

$$\left\{\begin{array}{l} f\left(X\right)=\overset{n}{\underset{i=1}{\sum }}\frac{f^{\left(n\right)}\left(X_{0}\right){\left(X-X_{0}\right)}^{n}}{n!}+\frac{f^{\left(n+1\right)}\left(X_{0}\right){\left(X-X_{0}\right)}^{\left(n+1\right)}}{\left(n+1\right)!}\\ h\left(X\right)=\overset{n}{\underset{i=1}{\sum }}\frac{h^{\left(n\right)}\left(X_{0}\right){\left(X-X_{0}\right)}^{n}}{n!}+\frac{h^{\left(n+1\right)}\left(X_{0}\right){\left(X-X_{0}\right)}^{\left(n+1\right)}}{\left(n+1\right)!} \end{array}\right..$$ (5)

So we need to take the derivative of *Z*. The first derivative of Z with respect to X can be written as follows:

$$\frac{dZ}{dX}=\frac{\partial Z}{\partial X}+\frac{\partial Z}{\partial Y}\frac{dY}{dX}$$

The second derivative of Z with respect to X can be written as follows:

$$\frac{d^{2}Z}{dX^{2}}=\frac{d}{dX}\left(\frac{\partial Z}{\partial X}+\frac{\partial Z}{\partial Y}\frac{dY}{dX}\right)=\frac{\partial^{2}Z}{\partial X^{2}}+\frac{\partial^{2}Z}{\partial Y\partial X}\frac{dY}{dX}+\left(\frac{\partial^{2}Z}{\partial X\partial Y}+\frac{\partial^{2}Z}{\partial Y^{2}}\frac{dY}{dX}\right)\frac{dY}{dX}+\frac{\partial Z}{\partial Y}\frac{d^{2}Y}{dX^{2}}.$$ (6)

The wild-type data can be viewed as a steady state of GRNs, only for as long as the flow of energy, nutrients, and other molecules is maintained. Hence, while the gene expression level of regulator *X* is at point $X_{0}$ , that is, the wild-type data of regulator *X*, the fluctuation of the gene expression level of targets *Y* and *Z* is minimal. This means the derivative of *Y* and *Z* with respect to *X* at point $X_{0}$ is zero. So we obtain the following equation:

$$\frac{dY}{dX}\left|X=X_{0}\right.=0,\frac{dZ}{dX}\left|X=X_{0}\right.=0,$$ (7)

that is,

$$f^{\prime }\left(X_{0}\right)=0,h^{\prime }\left(X_{0}\right)=0.$$ (8)

By substituting (7) into (6), we obtain the second derivative of Z with respect to X at point $X_{0}$ :

$$\frac{d^{2}Z}{dX^{2}}\left|X=X_{0}\right.=\frac{\partial^{2}Z}{\partial X^{2}}\left|X=X_{0}\right.+\frac{\partial Z}{\partial Y}\frac{d^{2}Y}{dX^{2}}\left|X=X_{0}\right.,$$ (9)

that is,

$$h^{\prime \prime }\left(X_{0}\right)=\frac{\partial^{2}Z}{\partial X^{2}}\left|X=X_{0}\right.+\frac{\partial Z}{\partial Y}\frac{d^{2}Y}{dX^{2}}\left|X=X_{0}\right..$$ (10)

For the value of ${\left(X-X_{0}\right)}^{n},n\geq 3$ is small enough, the terms of n≥3 in (5) can be neglected. So we can obtain an equation set:

$$\left\{\begin{array}{l} Y=f\left(X_{0}\right)+f^{\prime }\left(X_{0}\right)\left(X-X_{0}\right)+\frac{1}{2}f^{\prime \prime }\left(X_{0}\right){\left(X-X_{0}\right)}^{2}\\ Z=h\left(X_{0}\right)+h^{\prime }\left(X_{0}\right)\left(X-X_{0}\right)+\frac{1}{2}h^{\prime \prime }\left(X_{0}\right){\left(X-X_{0}\right)}^{2} \end{array}\right..$$ (11)

By substituting (8) into (11), we obtain the following equation:

$$\left\{\begin{array}{l} Y=f\left(X_{0}\right)+\frac{1}{2}f^{\prime \prime }\left(X_{0}\right){\left(X-X_{0}\right)}^{2}\\ Z=h\left(X_{0}\right)+\frac{1}{2}h^{\prime \prime }\left(X_{0}\right){\left(X-X_{0}\right)}^{2} \end{array}\right..$$ (12)

Due to $Y_{0}=f\left(X_{0}\right)$ and $Z_{0}=g\left(X_{0}\right)$ , (12) can be written as the following equation:

$$\left\{\begin{array}{l} Y-Y_{0}=\frac{1}{2}f^{\prime \prime }\left(X_{0}\right){\left(X-X_{0}\right)}^{2}\\ Z-Z_{0}=\frac{1}{2}h^{\prime \prime }\left(X_{0}\right){\left(X-X_{0}\right)}^{2} \end{array}\right..$$ (13)

Substituting (10) into the second equation in equation set (13), we obtain the following equation:

$$Z-Z_{0}=\frac{1}{2}\frac{\partial^{2}Z}{\partial X^{2}}\left|X=X_{0}\right.{\left(X-X_{0}\right)}^{2}+\frac{1}{2}\frac{\partial Z}{\partial Y}\frac{d^{2}Y}{dX^{2}}\left|X=X_{0}\right.{\left(X-X_{0}\right)}^{2}.$$ (14)

The first equation in Eq. 13 is equivalent to the following equation:

$$f^{\prime \prime }\left(X_{0}\right)=\frac{2\left(Y-Y_{0}\right)}{{\left(X-X_{0}\right)}^{2}},$$ (15)

that is,

$$\frac{d^{2}Y}{dX^{2}}\left|X=X_{0}\right.=\frac{2\left(Y-Y_{0}\right)}{{\left(X-X_{0}\right)}^{2}}.$$ (16)

By substituting (16) into (14), we obtain the following equation:

$$Z-Z_{0}=\frac{1}{2}\frac{\partial^{2}Z}{\partial X^{2}}\left|X=X_{0}\right.{\left(X-X_{0}\right)}^{2}+\frac{\partial Z}{\partial Y}\left|X=X_{0}\right.\left(Y-Y_{0}\right),$$ (17)

where $\frac{\partial^{2}Z}{\partial X^{2}}\left|X=X_{0}\right.$ and $\frac{\partial Z}{\partial Y}\left|X=X_{0}\right.$ are constants. Eq. 17 is a function for *Y* and *Z*. For simplicity, we set $z=Z-Z_{0}$ , $a=\frac{1}{2}\frac{\partial^{2}Z}{\partial X^{2}}\left|X=X_{0}\right.$ , $x={\left(X-X_{0}\right)}^{2}$ , $b=\frac{\partial Z}{\partial Y}\left|X=X_{0}\right.$ , and $y=Y-Y_{0}$ . Hence, Eq. 17 can be written as z=ax+by . We use multivariate linear regression to estimate the coefficients *a* and *b*, and then determine the causality between *Y* and *Z*.

On the contrary, we assume that gene *Z* regulates gene *Y*, and the gene–gene regulation can be expressed as a nonlinear equation set:

$$\left\{\begin{array}{l} Z=f\left(X\right)\\ Y=g\left(X,Z\right)=g\left(X,f\left(X\right)\right)=h\left(X\right) \end{array}\right..$$ (18)

Following the similar process to the aforementioned equation, we estimate parameter $\frac{\partial Y}{\partial Z}\left|X=X_{0}\right.$ to measure the relationship between *Z* and *Y*. Obviously, if gene *Y* truly regulates gene *Z*, then $\frac{\partial Y}{\partial Z}\left|X=X_{0}\right.=0$ will be the regression coefficient of linearization of model (17). Conversely, if gene Z truly regulates gene *Y*, then $\frac{\partial Z}{\partial Y}\left|X=X_{0}\right.=0$ will be the regression coefficient of model (18). Consequently, we compare the value of $\frac{\partial Z}{\partial Y}\left|X=X_{0}\right.$ with the value of $\frac{\partial Y}{\partial Z}\left|X=X_{0}\right.$ . If the value of $\frac{\partial Z}{\partial Y}\left|X=X_{0}\right.$ is larger than the value of $\frac{\partial Y}{\partial Z}\left|X=X_{0}\right.$ , it indicates that gene *Y* regulates gene *Z*. Conversely, if the value of $\frac{\partial Y}{\partial Z}\left|X=X_{0}\right.$ is larger than the value of $\frac{\partial Z}{\partial Y}\left|X=X_{0}\right.$ , it indicates that gene *Z* regulates gene *Y* (Figures 2A,B). With the iterative computation of gene $g_{i}$ and $g_{j}$ , the global network is constructed.

### Pseudocode of the DDTG algorithm

To describe the algorithm clearly, the pseudocode of the DDTG algorithm (see Algorithm 1) is provided in detail as follows:

Algorithm 1 DDTG.

 1: **Input:** Gene expression data *A.*
 2: **Output:** Inferred causal network *G.*
 3: **for** each gene *i* **do**
 4: Select the candidate downstream target genes for gene *i* based on matrix *S.* The number of the candidate genes is noted as *N.*
 5: Separate *N* candidate genes into two layers of the downstream targets by $I\left(g_{i},g_{j}|g_{k}\right)$ , that is, direct target set $\left\{g_{k}\right\}$ and indirect target set $\left\{g_{j}\right\}$ .
 6: Determine the causalities among the genes in the same layer for $\left\{g_{k}\right\}$ and $\left\{g_{j}\right\}$ by Taylor expansion.
 7: **end for**

## Result

In order to test the predictive power of the DDTG method, DREAM3 datasets (Prill et al., 2010) about *Yeast* knockout genes with sizes 10, 50, and 100 and two networks of size 10 from the DREAM4 datasets (Daniel et al., 2009) were used. The gold standard networks were generated with the nonlinear ODE systems in which the network structures were determined with detailed dynamics of both transcriptional and translational processes.

The predictive results were evaluated by following measures, that is, sensitivity or true-positive rate (TPR), false-positive rate (FPR), positive predictive value (PPV), accuracy (ACC), and Matthew’s coefficient constant (MCC). Mathematically, they are defined as the following expressions:

$$TPR=TP/\left(TP+FN\right),$$

$$FPR=FP/\left(FP+TN\right),$$

$$PPV=TP/\left(TP+FP\right),$$

$$ACC=\left(TP+TN\right)/\left(TP+FP+TN+FN\right),$$

$$MCC=\frac{\left(TP\cdot TN-FP\cdot FN\right)}{\sqrt{\left(TP+FP\right)\left(TP+FN\right)\left(TN+FP\right)\left(TN+FN\right)}},$$

where TP, FP, TN, and FN are the numbers of true positives, false positives, true negatives, and false negatives, respectively. TPR and FPR are also used to plot the receiver operating characteristic (ROC) curves, and the area under ROC curve (AUC) is calculated.

To validate the performance of DDTG, we compared it with several popular methods including LP (Wang et al., 2006), RO (Zhang, Liu, Liu, Duval, Richer, Zhao, Hao, and Chen, 2013), regression model-based LASSO (Geert et al., 2012), MI-based ARACNE (Margolin et al., 2006), and random forest-based GENIE3 (Van Anh et al., 2010), where the two alternatives with parameters “*sqrt*” and “*all*” in the GENIE3 were considered here, as they performed best in the DREAM challenges. For fair comparison, optimal default values of parameters from previous published articles were used in the running of these comparative algorithms. For example, regularization parameter λ of methods LP and RO were set to one, the ensemble parameter of method GENIE3 was set to 1,000, the threshold of MI filtering in method NARROMI was set to 0.05, and the threshold of MI in method DDTG was set to 0.1.

## Performance on DREAM3 benchmark data

DREAM3 datasets about *Yeast* knock-out genes with sizes 10, 50, and 100 were used.

**FIGURE 3:**
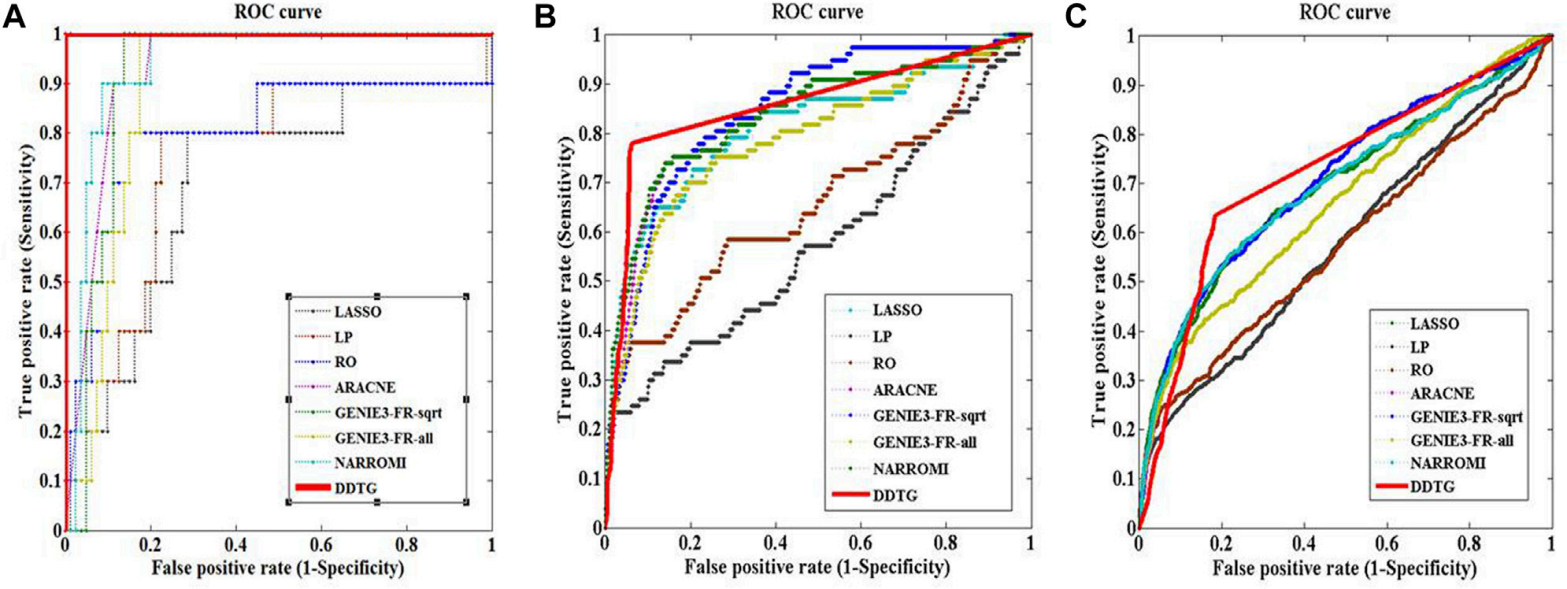
ROC curves of several methods on networks with different sizes. The solid line with star points is the ROC curve of method DDTG. **(A)** The ROC curves on the network with size 10. **(B)** The ROC curves on the network with size 50. **(C)** The ROC curves on the network with size 100.

First, DDTG was applied to the *Yeast* gene expression data with network sizes 10 and 10 samples. The comparison of DDTG with other methods is shown in Figure 3A, where DDTG outperforms other methods significantly with an AUC score of 1.000. From Figure 3A, we can see that all of the edges were detected. The performance of DDTG and other methods with respect to PPV, ACC, MCC, and AUC are shown in Table 1, where DDTG is superior to other methods.

**TABLE 1:** Comparison on networks with sizes 10, 50, and 100.

Method	TPR	FPR	PPV	ACC	MCC	AUC
Size 10						
LASSO	0.600	0.837	0.082	0.211	−0.191	0.703
LP	0.100	0.412	0.029	0.533	−0.202	0.738
RO	0.100	0.500	0.024	0.456	−0.252	0.798
ARACNE	0.900	0.112	0.500	0.888	0.618	0.930
GENIE3_FR_sqrt	0.700	0.112	0.437	0.867	0.483	0.919
GENIE3_FR_all	0.700	0.138	0.389	0.844	0.442	0.894
NARROMI	0.700	0.050	0.636	0.922	0.623	0.938
DDTG	**1.000**	**0.000**	**1.000**	**1.000**	**1.000**	**1.000**
Size 50						
LASSO	0.351	0.129	0.081	0.855	0.113	0.711
LP	0.389	0.085	0.130	0.899	0.182	0.669
RO	0.494	0.131	0.109	0.857	0.181	0.727
ARACNE	0.597	0.082	0.192	0.908	0.303	0.832
GENIE3_FR_sqrt	0.481	0.078	0.167	0.908	0.245	0.843
GENIE3_FR_all	0.442	0.073	0.164	0.912	0.231	0.796
NARROMI	0.532	**0.062**	0.217	0.925	0.307	0.839
DDTG	**0.779**	0.063	**0.284**	**0.931**	**0.445**	**0.856**
Size 100						
LASSO	0.403	0.112	0.175	0.861	0.199	0.696
LP	0.129	0.017	0.305	0.935	0.169	0.581
RO	0.245	0.056	0.206	0.906	0.175	0.580
ARACNE	0.118	0.016	0.304	0.936	0.161	0.695
GENIE3_FR_sqrt	0.007	**0.001**	0.308	**0.944**	0.040	0.710
GENIE3_FR_all	0.053	0.006	0.337	0.941	0.115	0.665
NARROMI	0.138	0.014	**0.364**	0.939	0.197	0.696
DDTG	**0.635**	0.185	0.169	0.805	**0.254**	**0.726**

The best performer for the relative item is noted in bold. LASSO, a regression method; LP, a linear programing-based method; RO, a recursive optimization-based method; ARACNE, a MI-based method; GENIE3, a random forest-based method; NARROMI, a method based on RO and MI; DDTG, a method based on dissecting the downstream target nodes.

Second, DDTG was applied to the *Yeast* gene expression data with network sizes 50 and 50 samples. The comparison of DDTG with other methods is shown in Figure 3B, where DDTG outperforms other methods significantly with an AUC score of 0.856. From Figure 3B, we can see that most of the edges were recovered. The performance of DDTG and other methods with respect to PPV, ACC, MCC, and AUC are shown in Table 1, where DDTG is superior to other methods.

Third, the *Yeast* gene expression data with network size 100 and 100 samples were used to evaluate DDTG and other methods. The ROC curves obtained by different methods are shown in Figure 3C, where DDTG outperforms other methods with an AUC score of 0.726. Table 1 shows the results obtained by different methods with respect to distinct performance indices. From the results, we can observe that DDTG performs better than most methods.

### Performance on DREAM4 benchmark data

The performance of network inference methods may strongly vary depending on the structural properties of the target networks. In order to assess the performance of DDTG predicting the topology on different target networks, DREAM4 datasets with size 10 were adopted here to evaluate our method.

While DREAM3 benchmarks were of a great value, there were some notable differences between DREAM3 and DREAM4 datasets. First, all the networks in DREAM3 were acyclic, while the networks considered in DREAM4 do contain cycles. Furthermore, a deterministic model was used in the DREAM3, while a stochastic one was used in DREAM4. Finally, both biological noise and experimental noise were added to DREAM4 datasets (Andrea et al., 2010). DREAM4 benchmarks consist of a set of networks with widely varying topologies. Two networks of size 10 from DREAM4 *in silico* challenge were adopted here to test our method.

**FIGURE 4:**
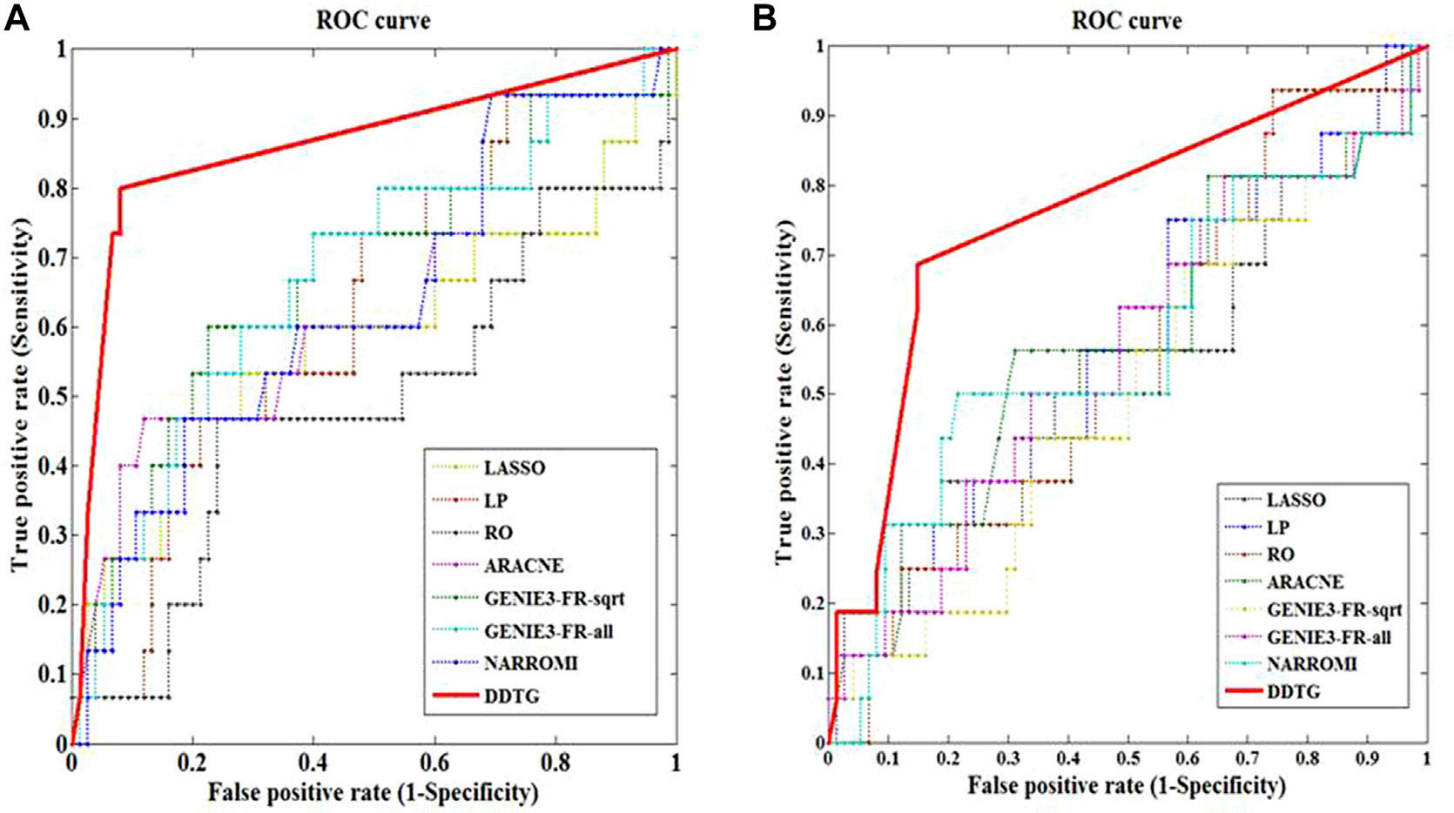
ROC curves of several methods on networks with different sizes. The solid line with star points is the ROC curve of method DDTG. **(A)** The ROC curves on network_1 from DREAM4 datasets with size 10. **(B)** The ROC curves on network_2 from DREAM4 datasets with size 10.

Figures 4A,B show the ROC curves by different methods on two different networks of size 10 from DREAM4 challenge. From figures, we can see that the performance of DDTG method is superior to that of other methods with the AUC values of 0.862 and 0.761. Table 2 summarizes the results obtained by different methods with respect to distinct performance indices. From Table 2, we can see that DDTG performs significantly better than other methods. Especially, when the DREAM4 datasets are used to test the performance of these methods, the accuracy of DDTG is still high. However, other methods except DDTG perform better in DREAM3 datasets; their performance decays rapidly in DREAM4 datasets.

**TABLE 2:** Comparison on networks from DREAM4 datasets with size 10.

Method	TPR	FPR	PPV	ACC	MCC	AUC
Size 10_1						
LASSO	0.533	0.720	0.129	0.322	−0.150	0.584
LP	0.467	0.240	0.280	0.711	0.189	0.627
RO	0.467	0.373	0.200	0.600	0.071	0.492
ARACNE	0.467	0.147	0.389	0.789	0.298	0.648
GENIE3_FR_sqrt	0.333	0.160	0.294	0.756	0.165	0.668
GENIE3_FR_all	0.333	0.147	0.313	0.767	0.182	0.667
NARROMI	0.333	0.120	0.357	0.789	0.219	0.630
DDTG	**0.800**	**0.080**	**0.667**	**0.900**	**0.671**	**0.862**
Size10_2						
LASSO	**0.813**	0.757	0.188	0.344	0.050	0.544
LP	0.250	0.297	0.154	0.622	-0.040	0.566
RO	0.375	0.378	0.177	0.578	-0.003	0.546
ARACNE	0.313	**0.149**	0.313	0.756	0.164	0.573
GENIE3_FR_sqrt	0.188	0.189	0.177	0.700	-0.002	0.501
GENIE3_FR_all	0.188	0.216	0.158	0.678	-0.027	0.558
NARROMI	0.313	**0.149**	0.313	0.756	0.164	0.573
DDTG	0.687	**0.149**	**0.500**	**0.822**	**0.479**	**0.761**

The best performer for the relative item is noted in bold. LASSO, a regression method; LP, a linear programing-based method; RO, a recursive optimization-based method; ARACNE, a MI-based method; GENIE3, a random forest-based method; NARROMI, a method based on RO and MI; DDTG, a method based on dissecting the downstream target nodes.

**FIGURE 5:**
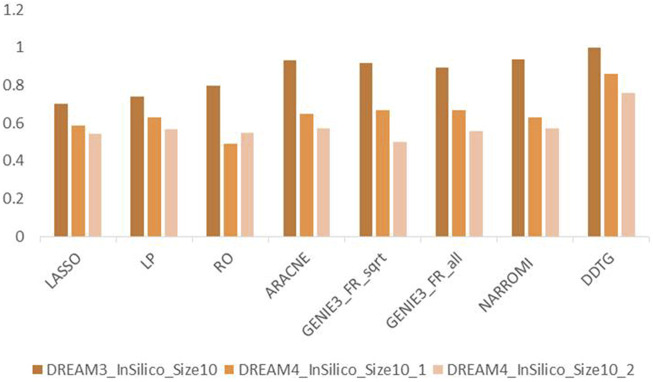
AUC comparison of methods on different networks with size 10.

Figure 5 shows the performance of the compared methods on DREAM3 datasets with size 10 and two networks from DREAM4 datasets with size 10. From Figure 5, we can find that the performance of DDTG varies less strongly than that of other methods in different datasets. It indicates that DDTG is more robust than other methods on different networks.

## Discussion

In this article, we proposed a novel method DDTG to reconstruct GRNs from gene knockout data. Yet our method can be applied to infer regulatory networks if gene knockdown or over-expression experiments are provided. This algorithm includes two steps. In the first step, the downstream targets are identified by comparing relative change values. In the second step, the hierarchy structure of the downstream targets is determined using CMI and MI. From the results, we can see that clearly DDTG is the best performer on the benchmark datasets. The good performance of DDTG may be contributed by following factors. Genes whose steady state values change after gene knockout can be immediately recognized by comparing the relative change values, which can improve the accuracy of network reconstruction. Meanwhile, due to the sparseness of GRNs, the downstream targets consist of a small number of nodes, which is helpful to reduce the redundant edges. Moreover, we assign a weight to the relative change values using sigmoid function. The parameters of the weight function depend on the expression level of each gene. This can reduce the noise for each gene (higher noise for a higher expression level). Therefore, other methods perform poorly in DREAM4 datasets, but the accuracy of DDTG is still high.

Furthermore, we construct gene–gene regulations using the *Taylor* formula at the steady-state levels of the wild type, and we use linear regression to determine the causal relationship between genes in the same layer for the first time. Finally, we infer the causal structure of GRNs using CMI and MI. Our method has the advantages of machine learning-based methods, such as making no explicit mechanistic assumptions and more computationally efficient.

Despite the advantages of DDTG, there are also limitations: DDTG is strongly dependent on the accuracy of identifying the downstream target nodes. The spurious downstream target nodes definitely result in spurious edges. For instance, the FPR by DDTG on datasets of size 100 is higher than that of other methods in Table 1. A technique to filter out and remove the impact of the spurious nodes may improve the performance of DDTG and will be considered in DDTG.

## Conclusion

We proposed a novel method, namely, DDTG, to improve the accuracy of GRN inference by dissecting the downstream target nodes. In this algorithm, the downstream targets for each gene are identified by comparing the relative change values. Furthermore, the causal structure of downstream targets is determined by CMI and MI. We especially use a weight function to reduce the noise for each regulator and determine the causality between nodes in the same layer using the Taylor formula and linear regression. The method was validated on the benchmark GRNs from DREAM challenge. The results confirmed the effectiveness of our method, which outperformed previous methods.

## Data Availability

The original contributions presented in the study are included in the article/Supplementary Material; further inquiries can be directed to the corresponding author.
